# Lectures on the Theory and Practice of Surgery

**Published:** 1846-01

**Authors:** 


### Lectures on the Theory and Practice of Surgery. By the late *Abraham Colles*, M.D. Edited by *Simon M'Coy*, Esq. F.R.C.S.I. Two Vols. 8vo. pp. 756. Dublin, 1845.

We have been accustomed to consider that one of the duties of an Editor of a work consisted in supplying it with a Preface; but the worthy publishers have taken the task upon themselves in the present instance, and favour us with an estimate of the value and peculiar merits of the "Lectures," which as might be expected, coming from such a source, is somewhat exaggerated. Merit, the work has, but it is going rather too far to characterize it as a "a Body of Surgery," suited to the requirements of the student and young practitioner, when important subjects (*e. g.* dislocations) are left wholly unnoticed, most others treated very superficially, and none completely. Why it is called "Lectures on the *Theory* and Practice of Surgery" we know not, since both publishers and editor rest much of its claim to notice upon the fact of the lecturer eschewing all notice whatever of prevalent theories, and his utter contempt for "writers on surgery," and "closet-surgeons," as contrasted with practical men. But we are disposed to think that an enlightened view of the principles which should guide us is no wise inconsistent with the delivery of due practical instruction, the result of opportunities we may have been favoured with. A great defect in these "Lectures" is the omission of all notice whatever of any of the modern improvements in surgery, and of the writings of the author's cotemporaries; and in fact, although they might have passed current some thirty years ago, when they were probably first delivered, they will not bear comparison with the complete courses of instruction our students are accustomed to at the present day, and should not be offered to them as such.

But although very defective as a "Body of Surgery," the Lectures contain many useful and even valuable observations, delivered with great candour, and with the confidence which Dr. Colles' very extensive practice fully justified. They indeed much resemble, both in the colloquial form of their delivery, and in their want of systematic completeness, the observations delivered at the present day by most surgeons to their pupils in their Clinical Lectures—not substitutes for, but invaluable adjuvants to, the more regular course of surgical instruction—in which the lecturer does not feel it incumbent upon him to follow the subject he is illustrating in all its details, but dwells upon those points especially to which his own attention has been devoted, or to which he thinks that of his hearers can be most beneficially directed.

We will now extract several of the useful practical observations which abound in the work.

*Opening Abscesses.*—"A rule has been laid down in books to open an abscess in the most depending part, but it is a bad rule, for that in fact may be the thickest part of the wall of the abscess, and you are only to be guided in your choice of the best part to make your puncture, by selecting the *thinnest* part of its parietes. If you open it anywhere but in the thinnest part, what will the consequence be? Why, just this—that Nature will make her opening where she at first intended, and this, even though the opening the surgeon made be in the most depending part, and be discharging freely."

*Erysipelas.*—Delirium or Coma coming on *before* the local inflammation does not indicate danger; but this is not the case when the eruption accompanied by slight constitutional symptoms has already continued for two or three days. When the tongue is brown, dry, and hard, the fever going on, the case is very dangerous. Hardness about the inflamed part is the best symptom for distinguishing phlegmon from erysipelas. When there is a great deal of pain present, vesicles are very apt to degenerate into foul or gangrenous ulcers. "You will find it laid down in books, that when erysipelas ends fatally, it is by its receding from the surface to some internal part. Now this is never the case. I have examined several after death who died of this disease, and have spoken to others who made similar investigations, and never saw any thing to bear out this opinion myself, nor heard it from any one who did witness an instance of it." The full, hard, and rapid pulse with irritability of stomach at the beginning of erysipelas, is not usually advantageously met by bleeding, but by causing free vomiting with tartar emetic, and, after the stomach has been well cleared, continuing it in smaller diaphoretic doses.

Purgatives are likewise very useful. In the typhoid form of the disease bark is not of much use, and many cases said to be cured by it, were in fact instances of rheumatism, mistaken for erysipelas. Partial redness appearing simultaneously during ordinary fever, in remote parts of the body, has been also mistaken for erysipelas; but true erysipelas never appears in different parts at the same time; and, whenever the redness is even contiguous, but not continuous, it is not erysipelas.

"On the going off of erysipelas, it sometimes leaves a fulness behind it, which pits on pressure like anasarca, but which is readily distinguished from it by the thickened and roughish cuticle. Now it is a remarkable fact, that if the skin be left in this morbid condition, it will be attacked a second time with erysipelas at some future period—it may be in three, six, or twelve months, but it will not escape another attack, one that will be more severe than the first, and which will leave a still greater fulness and thickness than the first did. When this happens, the patient will, in my opinion, be subject to returns of the malady for the rest of his life. Such a disease I should be inclined to call Chronic Erysipelas. I have tried every thing I could think of to cure this chronic form, and although in every trial I produced an amendment, I never completely succeeded with the succeeding affection, or in preventing a return of the erysipelas in its more acute form."

*Mammary Abscess.*—Abscess occurring after parturition produces hectic fever with great rapidity: but this disappears as soon as the matter is discharged; although in some cases it comes on even after this has taken place. Local treatment is of little or no avail, and active saline cathartics are the best medicines that can be given. If the abscess is opened early, much local irritation and profuse night-sweats are the consequence. Even when it is ripe it is better not to open it, unless, indeed, the matter is just under the cuticle; "but in general you ought to avoid the lancet, for if there be any life or thickness in the parts you cut through, you excite fresh inflammation, and greatly increase the chances of other abscesses." Whether opened or not there will usually be more than one abscess. Sometimes the inflammation, after threatening suppuration even for as long as ten days, is terminated by resolution. When the patient's health is bad, immense improvement results from a change of air: and the local hardness which is left is most effectually removed by sea-bathing. The wound left after the discharge of the matter frequently gives rise to fistulæ. These must all be laid completely open into one, as, if any are omitted, the operation is ineffectual. "If in this proceeding there should be a small portion of the mammary gland insulated, you may as well remove it entirely; it can never be of any use as a secreting organ, and may, by its presence, retard the healing of the remainder."

We cannot approve of Dr. Colles practice in these cases. A prompt opening of the abscess as soon as the existence of matter can be ascertained certainly much abridges the duration of the sufferings of the patient, and renders the formation of fistulæ, requiring so severe a proceeding for their removal, much less likely.

*Contusion of the Scalp.*—"Now you read that the bloody tumours of the scalp may be mistaken for a *depressed fracture*. Why, by a careless or superficial examiner it may be so. If you press the centre of the tumour with the point of the finger it will yield, and you think you can feel the edges of a circular depression of the bone, and you are told you are to distinguish them in this way. Run your finger along the scalp towards the tumour, and before it gets to its soft yielding centre it will have to rise over a ridge round its margin, and then it will suddenly sink, which would not occur if it were really a depression in the bone; but there is a better and more obvious method to distinguish one from the other. If the portion of the bone which receives the blow be really depressed, you will always find that the scalp is depressed along with it, and there will be no tumour at all; and, on running your finger over the place you feel the depression in the scalp, and the finger will sink *gradually* into the depression."

Death from *Erysipelas of the Scalp* has been usually attributed to a metastasis of the inflammation to some part within the cranium; but Dr. Colles states, that both he and Mr. Wilmot examined the bodies of several patients, in order to investigate this point, without ever finding any traces of inflammation of the brain or its membranes. There is nothing peculiar in the treatment of the disease when it is seated in the scalp—free vomiting by tartar-emetic, and sufficient purging, being the most successful means of relieving it. When the disease has quite disappeared the head should be shaved two or three times, as, if the hair is allowed to fall off of itself, it may not be reproduced.

*Concussion.*—A most fatal sign is furnished, when the respiration continues slow, while the pulse is rapid. " I never knew a patient under those circumstances recover, in whom the number of respirations and pulsations had not the natural proportion to each other." Dr. Colles does not believe we are in possession of any means of distinguishing symptoms which arise from concussion from those which are produced by compression. When we proceed to bleed the patient on his recovery from the first shock, we must always employ two stout persons to hold him, as, although he appears quite insensible, the instant he feels the prick of the lancet he starts forward with great force. The pulse is often very variable, and when beating but at sixty while the patient is lying down, it may be increased to 120 if he sits up or uses slight exertion.

" It will sometimes happen, that although when a patient is taken up after receiving the injury, he seems to have hardly any life in him, yet, by and bye, he begins to mutter, and after a little time he becomes perfectly delirious; but he has a method in the midst of the delirium. If left to himself, he will perhaps get up and dress himself; if he wants to make water, he goes regularly and looks for the proper vessel, and uses it like any other man in his sober senses. His pulse is, however, very quick; his movements unsteady; his eyes are morbidly acute. Now, I consider this a much worse case than when the patient is thrown into insensibility. I have, of my own knowledge, observed this delirious state to occur but in *extravasation*, but I believe it also occurs in concussion."

*Tumours of the Eyelids.*—These are not so easily removed as their great mobility would seem to indicate. When they are placed nearest the palpebral conjunctiva, they may be removed by dividing it, and thus a scar in the skin be avoided. " To know whether one of them is in front or behind the orbicularis muscle, all you have to do is to watch when the child cries, or to get the older patient to throw that muscle into action; and if it be next the skin the tumour will be only made more prominent, but if deeply seated, it will be flattened by being compressed between the ball of the eye and the orbicularis."

*Abscess of the Pharynx.*—"There is a case which sometimes requires bronchotomy. It is this. A patient comes to you and complains of a sore-throat and great difficulty of breathing; you examine his throat, and you see the tonsils in a natural state, as is likewise the velum palati, but look at the back of the pharynx, and you see its lining membrane protruded forwards. If you put in your finger, and press on this, you feel a softness, a want of resistance in the tumour—this is an abscess of the pharynx. Now, I have seen an abscess of this kind so large as to hold a quart of matter; I opened it with a lancet, and, although the patient was leaning forward, he was nearly suffocated with the sudden gush of matter. Such an abscess as this I would recommend you always to open with a flat trocar.

"In other cases, there appears to me a better operation than opening the trachea, and that is to get a common gum-catheter, cut off the end, leaving the eyes of the instrument on it, and introducing this through the nares into the larynx. There is no difficulty in doing this where we can get it through the nose, but there are some people who could not bear the instrument to be passed through the nose, and in such we must pass it through the mouth. Now, the great difficulty is to know whether the instrument has really entered the larynx or the œsophagus; you are told that you know this at once by finding whether air comes through the instrument or not—but air may come from the stomach as well as from the lungs. The way to know it beyond all doubt is, that if it has entered the larynx there will be a frightful convulsive cough and gasping at the moment of its entrance—you would really think the patient would die on the instant. But rest a few seconds, and this will gradually lessen, and at last subside, and the patient will afterwards bear the catheter, although with considerable distress; unless you pass the instrument through the nose, you can hardly, even with this test, be *quite* certain that it is in the larynx. * * * * * In introducing a catheter into the larynx, whether to allow the patient to breathe in such cases as I have spoken of, or for the purpose of inflating the lungs in persons apparently drowned, for instance, but particularly in the latter case, you will materially assist the furtherance of your object by pressing back the cricoid cartilage rather firmly against the bodies of the vertebræ, because by this manœuvre you close the orifice of the œsophagus: you will also draw forward the tongue, which will leave an uninterrupted and more direct course for your instrument into the larynx. If you require to pass a tube down through the œsophagus you will do the reverse of this—namely, to make the patient keep his tongue back, or if he be unable, in his agitation, to comprehend you, to press it gently back yourself, by which the epiglottis and root of the tongue will protect the larynx, and make the road to the œsophagus more direct."

*Compressing the Temporal Artery.*—Too forcible pressure is often employed for compressing the wounded temporal or other superficial artery lying on a resisting part. In consequence, ulceration and secondary hæmorrhage may be produced. The artery is seen pulsating violently, and it is erroneously believed that nothing but very strong compression will prevent bleeding, whereas a graduated linen compress, fastened down by sticking-plaster, and perhaps a light roller, suffices. Whenever it is possible, the compress must be placed over the skin, as then the delay consequent upon the filling up of the wound, and the production of a large cicatrix, are avoided. When, however, it is desired to apply pressure to the artery itself, a small piece of sponge or agaric is to be inserted, and allowed to make its way out of the wound of its own accord. Sometimes the little plug is found to be long held fast within the wound, notwithstanding this may be suppurating: the granulations having penetrated into the pores of the material employed. To prevent this, the sponge should be covered by a piece of fine linen before introducing it.

*Wounds of Arteries.*—Dr. Colles wisely omits no opportunity of placing before the young surgeon an account of the difficulties he will have to contend with; and thus graphically describes some of those attendant upon the taking up of wounded arteries.

"Sometimes the wound of the artery is not within two or three inches of the external wound. Suppose a man gets a stab of a knife in the arm, the knife runs up two or three inches, and then wounds the vessel—what are we to do in that case? Why, we are told the case is very simple—that all we have to do is to thrust a probe into the wound, and that it will of course guide us to the injured vessel, when we can slit up the intermediate parts, get at the artery, and take it up! No such thing—the probe will, in fact, run in any direction in which it is pushed, and will not lead to the artery at all except by mere chance. But, suppose it does lead to the spot where the artery is, you think, of course, it would be a very easy matter to take it up and tie it; it is not. We may talk as we please about our fine operations, but I protest I do not think in all surgery there is an operation *half* so difficult as taking up a wounded artery. You look and you see the blood coming, and you think you are just at the wounded spot of the vessel; but, perhaps, the artery is wounded an inch, or an inch and a half, or two inches from where you see the blood issuing, and the difficulty is increased if the operation is delayed for eight or ten days: you think you are well acquainted with the relative situation of the parts, and on cutting down you are surprised not to find the artery. Although you have two inches of it exposed you can neither see nor feel it pulsate: one of your assistants will cry out that he has it, but no one can feel it but himself, and it turns out that it was the pulsation of the artery in his own finger, which he mistook for that of the artery. Another thinks he can see the pulsation, but no one else can. You have no conception of the difficulties of the case. If you will make up your mind that you will not find the artery so superficial as you might, from mere anatomical recollections, expect, you will get rid of one of the causes of embarrassment; take your time, and you will get rid of another. It seems very plausible to say, in looking for a wounded artery, follow the track of the wound in the part, and you *must* come at length to the vessel, but it is very difficult even to trace the course of the wound; the cellular substance is everywhere stuffed with blood—nerves, arteries, tendons, and muscles, are all of the same colour; they are all out of their position; you lose your way among them, and the only way of finding it again is by dividing fresh parts; even when you are directly upon the wound of the vessel, you will not recognize it; for the contact of the air will prevent it bleeding in many instances."

*Retention of Urine.*—Among the causes of this, enumerated by Dr. Colles, is a *diminished sensibility of the bladder*, occurring in elderly persons, especially such as are engaged in sedentary or studious pursuits. After imperfect evacuation of the contents of the bladder has existed for some time, the patient, usually after a long sleep, finds himself unable to void his urine at all. Sometimes, after a few hours, some urine dribbles away, and continues to do so; and, as little pain is felt from the distension, advice is sought not for the purpose of relieving the retention, but for that of enabling the patient to *keep* his water. And yet, if the bladder be not emptied, the patient will be seized with fatal urinary fever. This is attended with its cold, hot, and profuse sweating stages, just like an ague, but differs from this in the irregularity with which the paroxysms come on. Patients suffering from urinary fever are said to emit an urinous odour from the skin and breath; but such is not the fact, such odour never occurring, unless some urine dribbles away and impregnates the bed-clothes, &c. In slighter degrees of urinary disease the disposition to this fever is shown by the great ease with which the patient becomes chilled. Blisters and other remedies proposed for this form of retention are of no use, the proper treatment being the passage of a catheter every six or eight hours. There is a form of retention to be carefully distinguished from this. It likewise occurs in the aged, and consists in very frequent passing of a spoonful or two of urine, which, however, eases the patient for a time. On placing the hand on the pubis you find no tumour, the bladder being very contracted in size. If, in such a case, you once use a catheter, the patient will never again do without it as long as he lives. You should keep him quiet, and upon moderate diet for a few days, and the irritation will subside. Demulcent drinks, as oatmeal tea, and honey, may be given, and, if there is much pain, an opiate suppository or enema should be employed.

In allusion to the difficulties in the detection of retention, Dr. Colles observes:—

"I mentioned but very cursorily how you were to know when there was really retention of urine; why, to read books written upon the subject, one would think there was nothing more easy. Now, I think, that sometimes there is nothing in surgery more difficult. You are told that all you have to do is to lay your hand above the pubis, and you will feel there the tumour of the distended bladder; and, to make the matter quite sure, you have only to introduce your finger into the rectum, and you will feel the fluctuation in the bladder pressing against it; but this is all nonsense. If a man gets an injury of the spine, with paralysis of the bladder, you will certainly have these tokens of the fact, and if you have a long finger, and there be no disease of the prostate gland, you may sometimes be able to feel the distension of the bladder through the rectum: but this is the very case where such proofs of retention of urine are not at all required, for you see what has happened, and you know the consequence; but it is in the *diseased* bladder, where the coats are thickened and contracted, that nothing can be felt to indicate retention. If, after death, you take out the bladder of such a person, and lay it on the table before you full of water, you will not be able to feel a fluctuation, let alone through the rectum, where, besides the additional thickness of the parts you have to feel so delicate a thing as fluctuation through, you have no means to make with proper effect a counter-resistance to your finger in the rectum, and the same fallacy would follow such trial as if you had tried to ascertain the existence of a collection of matter anywhere by feeling the part with one finger alone. But, besides this, the contraction of the sphincter on your finger is so strong, that it deadens its sensation, and you can feel nothing distinctly. * * * * * * I think you will always know in these cases that there is retention by passing your finger down along the linea alba, and when you come to the symphisis, you will feel just above it a hard, small, lump, just the top of the diseased bladder, containing urine, and retaining it, although much contracted. I have not had an opportunity of trying this, however, since it occurred to me."

*Fistula in Ano.*—Although this is usually found in the aged, sedentary, or diseased subject, it is at other times met with in young, active, and robust persons. When the abscess forms which is to lead to fistula, it must be promptly opened, without waiting for its pointing, or the cellular tissue about the rectum will become extensively implicated. When the abscess occurs in unhealthy subjects, it is sometimes very slow in its progress, occupying perhaps a month, and exhibiting a hard swelling, but no redness, and, what is strange, not manifesting a tendency to spread. These persons are commonly the subjects of disease of the lungs, the progress of which is delayed by the formation of the abscess about the rectum. Observing this, Dr. Colles has sometimes endeavoured to maintain a discharge from these fistulæ, but he has been seldom able to keep up any such derivative effect. When a complete fistula is ascertained to exist, there is no other means of treatment, upon which we should place the least reliance, than its complete division through the gut; but, if we find the patient is the subject of a grave pulmonary affection, we must decline operating. There are also local reasons for doing so.

"If, on examining the fistula with a probe, you find that it extends very high up—that is beyond the length of your finger introduced into the rectum, you should not operate, because you would probably wound the trunk of one of the hæmorrhoidal arteries, before it divides into branches, or some considerable venous trunk, and a hæmorrhage be the result, attended with some danger to the patient, and often very great difficulty and trouble to the surgeon to suppress it. If the course of the fistulous canal be not parallel to that of the rectum, that is, if, suppose, the upper part of the canal be a considerable distance from the rectum, and its lower part near it, or *vice versâ*, you should not operate; if the fistulous canal be parallel to the rectum, and that on passing a probe up into it you find every part of the canal even an inch distant from the coats of the gut, as felt by your finger in the latter, that distance is no objection to the operation. In making these examinations of the disease, you should observe one thing—not to introduce the finger into the rectum first, and then the probe into the fistula, but introduce the probe first, and then your finger; for, if you should distend the gut with your finger, you may not be able to trace the exact course of the fistula afterwards with the probe. In introducing a probe into a fistulous canal, take care that it is really passing in the course of that canal, otherwise you may be pushing it through the cellular substance in a wrong direction; in fact, little force will make it go in any direction as well as the right one."

When the knife has entered the gut, great force will be required, if the surgeon in cutting out merely *pulls* it towards him. He should employ a *sawing* motion, keeping the point of the bistoury against the point of his finger in the rectum. In dressing the wound with dry lint, this should be introduced on the probe first into the rectum, and pushed laterally from thence into the sinus, for by pushing it in at the wound itself, we can never be certain it has reached the bottom of the cavity.

*Fissure of the Rectum.*—This occurs in patients otherwise healthy, and is manifested by no external appearance. On examination, the rectum feels at first healthy, but "on closer investigation you will find a fissure, perhaps not half an inch long, in the mucous membrane of the gut, and extending in depth only through this membrane, and it may be seated at the back, or at either side of the bowel." There is a symptom, however, which indicates this disease even more certainly than an examination of the rectum itself, viz. the existence of a distinct interval of from ten minutes to one or more hours, between the passage of a stool and the production of a most violent, burning, pain. Boyer's procedure of dividing the rectum through the fissure gives effectual relief, no dressing of any kind being required. Dr. Colles has relieved cases, where this operation was declined, by rubbing lunar-caustic on the part affected.

*Syphilitic White-Swelling.*—"There is a form of white-swelling of the knee which I wish particularly to call your attention to, as the distinguishing it from others is absolutely necessary to successful treatment, for its nature is totally different from all others. It is this: patients who have secondary syphilis will often get a pain and effusion into the knee, like the white-swelling of children, and it is a true and perfect symptom of the venereal disease in the secondary form. A pupil of mine saw a case of this kind in a hospital in London, and mentioned to Sir A. Cooper what he thought it was. Sir Astley said he was mistaken, but that, to satisfy him, he would give the patient mercury—he did so—the knee got well under it—and Sir Astley himself declared it was the first case of the kind he had ever seen. This case may be distinguished from common white-swelling by one remarkable symptom—viz. the popliteal space is not filled up, as it always is in white-swelling. In the acute cases of white-swelling you will cure nine cases out of ten by throwing in calomel as quickly as possible."

### *The Venereal Disease.*

The Lectures relating to this subject are the best in the work. Dr. Colles is a determined advocate of the mercurial treatment of syphilis and its sequelæ, and delivers many important practical precepts for the due regulation of this. Before noticing some of these we may extract an observation or two upon

*Gonorrhœa.*—Discharges produced by stricture, or urinary disease, are often confounded with this, and are chiefly to be distinguished from it by the fact of their coming on much sooner after connexion than gonorrhœa does, and unsucceeded by inflammatory symptoms. Persons liable to gout or rheumatism may have urethral discharges also, not to be distinguished from a gonorrhœa. Dr. Colles believes that clap is by no means so common a disease as it is generally supposed to be; and that chancres are much more frequently met with. Injections are very often useless from the inefficient manner in which they are employed. The point of the syringe should be introduced far into the urethra, the fluid slowly injected, and the instrument not withdrawn until some time afterwards—the fear of the injection penetrating too low down, being quite groundless. In most cases, two syringe-fulls thrown in three times a day suffice. Although low diet, aperients, and repose are adapted for the majority of cases, some subjects, in whom the discharge is usually thin and unhealthy, will thrive best upon a more generous regimen. *Chordee* is often kept up by other causes than clap, as stricture, &c. When seized by it, the patient may be relieved by getting out of bed and placing himself on his elbows and knees, when the chordee stops at once. There is sometimes considerable hæmorrhage from the urethra, which excites unnecessary alarm, as it is frequently beneficial. When *both* testes are morbidly sensitive, so as not to bear the slightest touch, the affection is purely sympathetic, and hernia humoralis need not be feared; but this is to be expected when the uneasiness proceeds along the chord, and is confined to one side. Hernia humoralis arises more from constitutional causes than local extension, and is preceded by fever. An emetic given quite early and attention to diet will cut short the disease, which rarely appears during the inflammatory stage of gonorrhœa, and never attacks both testes at once, although these may become alternately affected, even two or three times. Sometimes gonorrhœa gives rise to most distressing irritability of the bladder, and then to complete retention. Hip-baths and anodyne enemata must be had recourse to, but, if by these means you cannot give speedy relief, the catheter must be used, for, if this be delayed too long, the patient will ever after be unable to pass water without an instrument. A large one is introduced with most ease. The practice of stimulating the urethra to reproduce the discharge, which has become suppressed by the febrile action preceding hernia humoralis, is a very useless one.

*Venereal Hectic from the Non-Mercurial Treatment of Syphilis.*—Dr. Colles does not deny that genuine syphilitic sores may be healed without mercury, but truly observes that, supposing this were the most eligible proceeding, which is not the case, the consumption of time, and the necessity for the confinement of the patient, would render its adoption in private practice impossible. But, even where these requisites can be fulfilled, the patient is often no gainer.

"A man who has had his chancre cured without mercury has sometimes a hard knob or tumour remaining where the original chancre had been, sometimes as large as a filbert, of a bluish colour, and without pain. It gives him no trouble—but there it remains; now, in such a case, you will find that, from the time the chancre is healed, his health begins to decline—he falls into a hectic state. Such a case came across me in a state so far advanced in hectic that I thought he was beyond all aid. I decided at once the man was labouring under syphilis. I gave him mercury, and in eight or ten days he was at least safe, and finally recovered perfectly under the mercurial treatment. * * * You will not succeed in relieving these cases by trifling, you must give mercury at once, although cautiously, and in small doses. I have seen some more of these cases, and some closely resembling them, which followed an imperfect cure by mercury, carelessly or injudiciously exhibited; these cases are, however, rarely met with. Another man gets his chancre healed without mercury, and (as soldiers have been the subjects of these trials) he is thought only to be evading his duty when he complains to the surgeon, and, as a military surgeon informed me, he gets into a state of constitution that confounds all previous knowledge of disease.

"In some of these cases we shall have a man with both primary and secondary symptoms existing at the same time—with febrile action going on either of the inflammatory or typhoid type, of the irritative or hectic kind—with all the animal powers reduced to the lowest ebb—and perhaps an eruption coming out at the same time on the skin, to which a due share of the fever may be attributed, but altogether in a most anomalous and deplorable condition. To venture on mercury in such a case, can be only from the experience that nothing else seems to produce much effect, and after the first trial, from seeing its success."

In such cases Dr. Colles employs the remedy very cautiously, rubbing in only ten or twelve grains of the *ung. hyd.* or giving three or four grs. of

*pil. hyd.* every night, prescribing bark at the same time when the system is much depressed.

*Modes of Administering Mercury.*—Dr. Colles believes *inunction* to be the best means of affecting the system. The quantity to be used at one rubbing should be divided into three or four portions, and each well rubbed in before another is began with. The friction should be performed by another person, the patient not being usually competent to the requisite exertion. If the patient cannot remain within doors, mercury must then be exhibited internally, when, however, it is liable to act on the bowels. To prevent this, we must not be in too great a hurry to add opium, as frequently in two or three days the irritation will subside of its own accord, and not return unless the preparation given be changed, or the dose too much increased. Calomel affects the mouth more quickly than other preparations, but is more apt also to act on the bowels if it be not combined with opium. Corrosive sublimate causes the rapid disappearance of some of the secondary symptoms, but the cure effected by it is not radical unless it be followed by some of the other preparations. Under favourable circumstances the mercury should manifest its effects in a week or ten days, and, although there are great differences in this respect, if no effect be produced at the end of a fortnight, the mercury should be stopped for a day or two, the patient purged, and a couple of warm-baths given him, when the mouth will frequently become sore, although the mercury has not been re-commenced. If the system resists the mercury we must not endeavour to produce the effect by increasing the quantity given, but examine the condition of the health, and thus, if we find fever existing, as indicated by a *dry* mouth, we must suspend the drug until this is relieved.

"There is nothing more important to remark than the condition of the gums under the use of mercury, and the degree of salivation produced. The gums may swell and ulcerate, and yet the mercury is disagreeing with the constitution, and doing no service to the disease. If the gums swell, remaining red however, and a salivation comes on, all is right; but, if it causes *ulceration* of the gums—if the gums are receding from the teeth, and that there is *no discharge of saliva*, mercury is doing no good, but mischief. If you continue to use it without this effect being produced, you must do one of two things—either double the dose of mercury, and by so doing, when you least expect it, throw your patient suddenly into a profuse salivation, which you will not perhaps be enabled to control; or you must retrace your steps, and alter the constitution by attending to the bowels, or doing whatever else seems indicated in the particular case, and when this is done, a proper salivation will often be the consequence, without another particle of medicine being given. * * * * Now, mercury, from peculiarities of constitution, cannot be made to affect the gums of all, and in some of these cases it will affect the throat instead. About the usual period it should be expected to show itself, the patient will experience some uneasy feeling in the throat, and, on your examining it, the soft palate will be found thickened and red, and an ash-coloured slough on one or both of the tonsils. This would spread rapidly if the mercury were rashly pushed without care and attention to the constitution. Although this is a local demonstration of the action of mercury, it is not one on which I should wish to place much reliance as a curative for the venereal disease; you must sometimes, however, be content with it, as no other can be had, and I must admit I have cured the primary disease without other local effect of mercury."

Dr. Colles does not advocate the production of anything beyond a moderate action on the mouth, which, however, is to be maintained until all induration has disappeared. The endeavouring to force mercurialization upon systems which are little susceptible of its influence by means of very large doses, may give rise to a profuse salivation, without the consolation of forwarding the progress of the case. When we are aware of this peculiarity in a case, we should commence treating it by *small* doses. The chancre is sometimes healing under the mercury, when the treatment being impatiently urged on, the sore puts on a fungous or spreading character, when all mercury must be discontinued. When fever is developed during a mercurial course, too, the drug must be discontinued. If, after the mouth has began to be affected, the patient neglects himself, and allows the effect to subside, much more mercury will be necessary to reproduce it than was at first requisite to induce salivation.

*Bubo.*—It has been said that, if a chancre is healed up suddenly, a bubo will be caused ; but, in truth, this results from the too sudden and violent effect of the mercury upon the constitution, and furnishes an additional reason for the cautious employment of the drug. In proof of this, when the chancre is not doing so well and a bubo is threatening to form, if the mercury be suspended for a day or two, and a purgative given, the sore will recover its healthy appearance, the bubo diminish, and the patient perhaps recover without requiring any additional mercury. But a case may present itself in which no mercury has been used, and in which chancre and bubo are both present, and for this, mercury is the proper remedy, which must be employed even though the bubo threatens to suppurate, and may seem at first to be the worse for it. When, however, a bubo continues to get worse under the use of mercury, and induces fever, the medicine must be suspended. It is of very little consequence whether a bubo, which has began to point, is allowed to burst or is opened by a lancet ; but we must at all events never meddle with one that is hard and unripe. A bubo occurring in a person of weak, lax, habit, or in deranged health, and which remains indolent and flat, the skin over it being of a bluish or purple colour, requires to be opened by a large incision completely traversing this diseased skin. There is no advantage derivable from opening any description of bubo by means of caustic. When a bubo that has been opened forms painful fistulous canals towards the scrotum, mercury, which has been given in excess, must be suspended, the cavity of the ulcer stuffed with red precipitate, over which a pledget of tow is firmly bandaged, and measures taken which may contribute to the improvement of the general health. No benefit attends the division of these fistulæ.

*Venereal Eruptions.*—" You do not observe in syphilitic eruptions what is seen in other eruptions to which they have been thought somewhat analogous—namely, that the whole of the eruption comes out at once, that is within a few hours. In the syphilitic, you may see it declining in one part and appearing in another at the same time ; you very often have an opportunity of observing it in several stages of its progress at any time during the first month, until it all at last fades away, at no determinate period from its first appearance. Sometimes the eruption is extensively spread over the surface of the body, but at others it is confined to one region, or even to a spot you might cover with your hand. You might, perhaps, suppose that the more of the eruption that appeared the more mercury should be required for its removal—but, I think, the very reverse is the case. I think, where the eruption comes out fully and fairly, it yields more quickly and completely than where you have only a few spots here and there. Some suppose there is great advantage gained by treating these eruptions *early* with mercury; but I am far from an unconditional acquiescence in this notion. I think you may begin to prescribe mercury too soon, and for this reason; the eruption is ushered in with fever, and among many individuals in this state, you will see every degree of severity in the symptoms of this fever, from the most trifling to the most intense: but, whether mild or severe, your first duty is to remove the fever; for, if you give mercury during the eruptive fever, you will not do the eruption one bit of good, but will probably do the patient a mischief."

Mercury in large doses does much harm in these syphilitic eruptions; and Dr. Colles states, that they are cured with great rapidity by rubbing in from 10 to 20 grs. of *ung. hyd.* or giving from 3 to 5 grs. of pil. hyd. every night, either alone or with tonics, or mild diaphoretics. The scaly eruptions fade quickest under this treatment, then the papular, and the copper-coloured blotch. Rupia will, however, not tolerate the employment of mercury; and the pustular eruptions require it to be used in the most cautious manner. In cases where the mercury acts beneficially, it should be continued for at least six weeks, or the eruption will be reproduced, although in a more manageable form. When an eruption is situated in the face, the disfigurement may be most speedily removed by giving corrosive sublimate; but the treatment must be finished by friction or blue-pill, or some other secondary symptom will follow.

*Syphilis combined with Scrofula.*—Dr. Colles has many interesting observations upon the modification which a scrofulous condition of the system impresses upon syphilis, and by no means agrees with those who prohibit the employment of mercury under such circumstances—having indeed seen some of the worst results spring from the non-mercurial treatment in these cases. We have only space to extract one or two of his remarks.

"So far am I from being afraid of inducing salivation in those complicated cases, that I am on the contrary desirous to bring it on quickly,—much more so than I would be eager to do in a common case, and by the practice I have witnessed the most beneficial results. I am not at all disposed to deny that if mercury, when given in these cases, should not exhibit itself in the proper manner in the gums and salivary glands, but that it may and probably will exasperate the superinduced scrofulous affection, but from what I have endeavoured to impress upon you concerning the salutary action of this drug in any case where salivation may be necessary, you may readily understand that our present case is but another example of a general rule, and not an exception to that rule, as many seem to consider it. What I do then in instances where the glands of the neck become affected through the venereal stimulus is, when I do not see the mercurial ointment or blue-pill affect the mouth quickly, I order calomel, with or without opium, to be added to the other form, and when the gums become touched, and some ptyalism produced, the secondary venereal symptoms rapidly recede, the swollen glands grow smaller, and if one of them has opened into an ulcer it takes on a healing disposition, and frequently cicatrizes by the time we judge enough of the medicine has been given to cure the venereal affection."

The preceding observations are applied especially to the case where the glandular affections are excited by the secondary venereal symptoms; but wherever the patient is under the full scrofulous action when he contracts the syphilis, there is no reason why full mercurial action should not be induced, and the syphilitic symptoms will yield as readily in this case as in a less complex one. So, too, even in patients threatened with phthisis, mercury must be cautiously given when they become the subjects of syphilis, provided that the case has not gone so far as to produce even slight hectic, when mercury will do harm.

*Secondary Venereal Ulcers.*—These, like primary ones, will sometimes heal before the constitution has been sufficiently impregnated with mercury, and if this be left off too soon, or irregularly administered, numerous and harassing relapses are sure to occur for months, or even years. Not only may the practitioner be deceived by the sore thus healing too speedily, but also by its seeming at first to get worse instead of better under the use of the drug. A feverish state of the system, or other causes of general disturbance, may give rise to this, and yet, when the mercury is cautiously, but effectively, continued, all is found to go on well. It requires, sometimes, great courage to persevere, in spite of apparent contradictions, but nothing is so mischievous in these cases as timid half-measures. It is not at the *beginning* of a mercurial course that we must stop the treatment on the first unfavourable appearance occurring, for this may arise from causes independent of the specific action of the medicine. If, however, after full action is established, a change for the worse occurs, it must be at once suspended.

The Lectures abound in useful practical remarks upon the diagnosis and treatment of the various secondary symptoms; but we have not space for further extracts. Dr. Colles certainly extends the application of mercury to many cases usually thought to forbid its employment; and, indeed, there hardly seems any case in which he does not think it may, by the aid of due modifications in the modes of its administration, be advantageously used. We believe that modern practitioners will judiciously prefer, in many of these secondary symptoms, having recourse to the *iodide of potassium* in full doses. We, however, cordially agree with the lecturer in his numerous protests against attempting to treat the primary symptoms of syphilis without mercury. Their removal may sometimes, though certainly not always, be accomplished: but where are the advantages gained, and yet how great are the risks incurred. In concluding our notice of the work, we may repeat the opinion we expressed at its commencement; that, although these "Lectures" are very imperfect, considered as a course of instruction upon Surgery, they contain much valuable practical matter.

